# Group‐based trajectory modeling of intracranial pressure in patients with acute brain injury: Results from multi‐center ICUs, 2008–2019

**DOI:** 10.1111/cns.13854

**Published:** 2022-05-25

**Authors:** Fan Yang, Chi Peng, Liwei Peng, Peng Wang, Chao Cheng, Wei Zuo, Lei Zhao, Zhichao Jin, Weixin Li

**Affiliations:** ^1^ Department of Plastic Surgery and Burns, Tangdu Hospital Fourth Military Medical University Xi'an China; ^2^ Department of Health Statistics Second Military Medical University Shanghai China; ^3^ Department of Neurosurgery, Tangdu Hospital Fourth Military Medical University Xi'an China

**Keywords:** clinical outcomes, group‐based trajectory modeling, hemorrhagic stroke, intracranial pressure monitoring, traumatic brain injury

## Abstract

**Objective:**

The objective of the study was to characterize the longitudinal, dynamic intracranial pressure (ICP) trajectory in acute brain injury (ABI) patients admitted to intensive care unit (ICU) and explore whether it added sights over traditional thresholds in predicting outcomes.

**Methods:**

ABI patients with ICP monitoring were identified from two public databases named Medical Information Mart for the Intensive Care (MIMIC)‐IV and eICU Collaborative Research Database (eICU‐CRD). Group‐based trajectory modeling (GBTM) was employed to identify 4‐h ICP trajectories in days 0–5 post‐ICU admission. Then, logistic regression was used to compare clinical outcomes across distinct groups. To further validate previously reported thresholds, we created the receiver operating characteristic (ROC) curve in our dataset.

**Results:**

A total of 810 eligible patients were ultimately enrolled in the study. GBTM analyses generated 6 distinct ICP trajectories, differing in the initial ICP, evolution pattern, and number/proportion of spikes >20/22 mmHg. Compared with patients in “the highest, declined then rose” trajectory, those belonging to the “lowest, stable,” “low, stable,” and “medium, stable” ICP trajectories were at lower risks of 30‐day mortality (odds ratio [OR] 0.04; 95% confidence interval [CI] 0.01, 0.21), (OR 0.04; 95% CI 0.01, 0.19), (OR 0.08; 95% CI 0.01, 0.42), respectively. ROC analysis demonstrated an unfavorable result, for example, 30‐day mortality in total cohort: an area under the curve (AUC): 0.528, sensitivity: 0.11, and specificity: 0.94.

**Conclusions:**

This study identified three ICP trajectories associated with elevated risk, three with reduced risks for mortality during ICU hospitalization. Notably, a fixed ICP threshold should not be applied to all kinds of patients. GBTM, a granular method for describing ICP evolution and their association with clinical outcomes, may add to the current knowledge in intracranial hypertension treatment.

## Graphical abstract

To summarize, a novel ICP trajectory that could enable us to move the treatment of ABI from a fixed threshold approach to a more individualized treatment was proposed. ICP values and variability differed across these six identified trajectory groups with favorable vs. unfavorable outcomes. The epidemiological shift toward a larger proportion of physiologically fragile elderly patients calls for more attention.

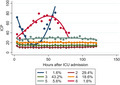

## 1 INTRODUCTION

Acute brain injury (ABI) is a catastrophic cerebrovascular event with high morbidity and mortality worldwide, with more than 14 million people estimated to live with disabilities related to ABI in the Europe and the USA.^1^, ^2^ ABI results in the activation of primary and secondary pathophysiological processes which may lead to a continued increase in intracranial pressure (ICP), followed by brain herniation, brain ischemia, and death if left untreated.^3^ To be specific, the pathogenesis of ABI included blood–brain barrier (BBB) disturbance, excitotoxicity, mitochondrial dysfunction, oxidative stress, inflammation, and apoptosis.^4^, ^5^ As a cornerstone of care in managing ABI, ICP monitoring facilitates the rapid assessment of the injuries, and further, institutes life‐saving protocols in a timely manner.^6^ However, several uncertainties remain.

First, the most recent Brain Trauma Foundation (BTF) guidelines suggested that ICP monitoring was used in the management of severe traumatic brain injury (TBI), but the influence of ICP monitoring on clinical outcomes was derived from low‐quality evidence.^7^ Moreover, the only two randomized controlled trial (RCT) assessing ABI management based on ICP monitoring demonstrated contradictory results.^8^, ^9^ Indeed, RCTs represent the highest level of evidence, but they often lack generalizability to real‐world patterns of care for carefully selected patient populations and regimented treatment protocols. Finally, although elevated ICPs are also common in patients with hemorrhagic stroke, few data are available to provide neurosurgeons with guidance on ICP management in this setting.^10^, ^11^, ^12^, ^13^

Current ICP management guidelines for hemorrhagic stroke including intracerebral hemorrhage and subarachnoid hemorrhage have been extrapolated from those for TBI.^14^ Furthermore, there is uncertainty concerning ICP treatment threshold. Historically, the most widely accepted ICP threshold for therapy was 20 mmHg, although the latest guidelines proposed a “new” threshold of 22 mmHg. Given the age and gender differences, the recommended threshold for older patients (≥ 55 years) and females was 18 mmHg.^7^ Nevertheless, the strength of evidence on this suggestion was questionable, owing to the fact that it stemmed from one single center retrospective observational study.^15^ More fundamentally, due to the fact that the pathophysiology of ABI is highly heterogeneous and dynamic, a one‐size‐fits‐all management strategy is unlikely to be the optimum. It may mask the substantial underlying effect of longitudinal ICP changes on clinical outcomes. More precise understanding of intracranial disturbances might identify patients at risk for unfavorable trajectories early, ultimately provide individualized targets and, hopefully, targeted therapies.

Group‐based trajectory model (GBTM), an established analytical approach, may provide an alternative methodology for summarizing long‐term ICP value while accounting for the dynamic nature of this variable over time.^16^, ^17^, ^18^ Through estimating the change over time for repeatedly measured outcomes, GBTM is able to identify distinct clusters of individuals who follow similar longitudinal response patterns. Based on finite mixture modeling of unobserved subpopulations, and hypotheses regarding trajectory shape, the number of diverse trajectory groups can be tested by maximum likelihood.^19^ The present study aimed to characterize ABI patients by their ICP status in intensive care unit (ICU), to explore whether GBTM added sights over conventional thresholds, and to analyze differences in clinical outcomes across defined groups.

## 2 METHODS

### 2.1 Data source and ethics approval

These two sizeable critical care databases, the Medical Information Mart for Intensive Care (MIMIC)‐IV version 1.0^20^ and eICU Collaborative Research Database (eICU‐CRD) version 1.2^21^ were employed for the study. Briefly, as an updated version of MIMIC‐III, the MIMIC‐IV database incorporated comprehensive, de‐identified data of patients admitted to the ICUs at the Beth Israel Deaconess Medical Center in Boston, Massachusetts, between 2008 and 2019, containing data from 383,220 distinct hospital admissions (single‐center). The other database, eICU‐CRD, was a multi‐center, sizeable database consisting of high‐quality data covering over 200,000 admissions to ICUs across the United States between 2014 and 2015. One author (CP) who has finished the Collaborative Institutional Training Initiative examination obtained access to both databases and was responsible for data extraction (Certification number: 41657645). Since the study was an analysis of the anonymized publicly available databases with pre‐existing institutional review board (IRB) approval from the Massachusetts Institute of Technology (MIT) and Beth Israel Deaconess Medical Center (BIDMC), informed consent was also waived. The study was reported in accordance with REporting of studies Conducted using Observational Routinely collected health Data (RECORD) statement.^22^

### 2.2 Study population

Inclusion criteria were patients with a diagnosis of TBI or acute brain injury due to intracranial hemorrhage or subarachnoid hemorrhage. People with Glasgow Coma Scale (GCS) > 12, age less than 16 years old, and those who stayed in ICU < 24 h were excluded from the study. Moreover, for patients with ICU admissions more than once, only data of the first ICU admission of the first hospitalization were included in the analysis. Of these, patients who had ICP recordings were ultimately included.

### 2.3 Data collection

In this study, detailed demographic data were collected on age, gender, race, body mass index (BMI), and smoking history. Coexisting disorders were also collected. Then, the Charlson comorbidity index (CCI) was calculated from its component variables. In addition, we extracted data containing type of injury (TBI, subarachnoid hemorrhage, and intracranial hemorrhage), mechanism of injury (unintentional falls, motor vehicle crashes, and firearm injuries), multiple scoring systems [GCS, sepsis‐related organ failure assessment (SOFA), acute physiology score III (APSIII), injury severity score (ISS)], medication, and neurosurgical interventions on the first day of ICU admission. For some variables recorded more than once within the first 24 h after ICU admission, the one associated with the highest acuity of illness was used. Outcomes were determined by all‐cause mortality (30‐day) and GCS value at discharge from ICU (Improvement: GCS > 12; No improvement: GCS ≤ 12). Favorable outcomes referred to the decrease in mortality and improvement in GCS.

### 2.4 Statistical analysis

Continuous variables were presented as median with interquartile range or mean with standard deviation, and categorical variables as total number and percentage. Proportions were compared using the chi‐square test or Fisher exact tests while continuous variables were compared using the t test (normally distributed) or Wilcoxon rank sum test (not normally distributed), as appropriate. Pairwise comparisons were adjusted (Bonferroni's method).

## 3 GBTM

### 3.1 Modeling of ICP evolution trajectories

GBTM assumes that the population is heterogeneous and composed of several classes of subjects characterized by a number of mean profiles of trajectories.^23^ Consistent with previous study,^24^ we applied GBTM to identify subgroups of subjects sharing distinct ICP courses within 120 h following ICU admission (Every patient has been tested every 4 h). Models were tested from 2 trajectory classes to 6 trajectory classes (shapes from linear, quadratic, and cubic). The best number of classes was evaluated by parameters such as average posterior probability (AvePP), estimated probability (%), odds of correct classification (Occ), Bayesian information criteria (BIC), Akaike information criterion (AIC), and Log‐likelihood (LL). The smallest BIC, AIC, and LL value accompanied by higher AvePP (≥0.7) and Occ will be selected.^25^

### 3.2 Effect of ICP trajectories on clinical outcomes

After identifying ICP evolution trajectory groups, we evaluated the associations between trajectory subgroup membership (as a categorical exposure) and clinical outcomes using logistic regression models. In these analyses, we assigned group 1 as the reference. Initially, a crude logistic model was constructed. Then, all models were adjusted for the minimum set of potential confounders, namely, age, sex, race, BMI, smoking history, CCI, type of injury, mechanism of injury, medication (mannitol use and hypertonic saline use), and surgical interventions [craniectomy, ventriculostomy, and cerebrospinal fluid (CSF) drainage].

### 3.3 Estimating trajectory membership

We performed multinomial logistic regression models to calculate adjusted odds for trajectory membership based on patient, clinician, and prescription characteristics. Models were adjusted for age, sex, race, BMI, smoking history, CCI, type of injury, mechanism of injury, medication, and surgical interventions. Besides, in order to verify the previously recommended ICP threshold in the 4th edition guidelines for TBI,^7^ we also performed receiver operating characteristic (ROC) curve analysis in this dataset.

### 3.4 Sensitivity analysis

As a sensitivity analysis, we developed an additional GBTM model including only elderly subjects (age ≥ 55 years) subjects and explored in‐hospital outcomes among this specific group with different characteristics. Moreover, the patterns assumed missing to be completely at random, so multiple imputation approach was used to iterate the original data on the first day of ICU admission (Table S1).^26^

Statistical significance was considered to be at two‐sided *p* < 0.05. All analyses were performed with R version 4.0.2 (http://www.R‐project.org) and STATA (version 16.0; Stata Corporation, College Station, TX, USA).

## 4 RESULTS

### 4.1 Study cohort

**FIGURE 1:**
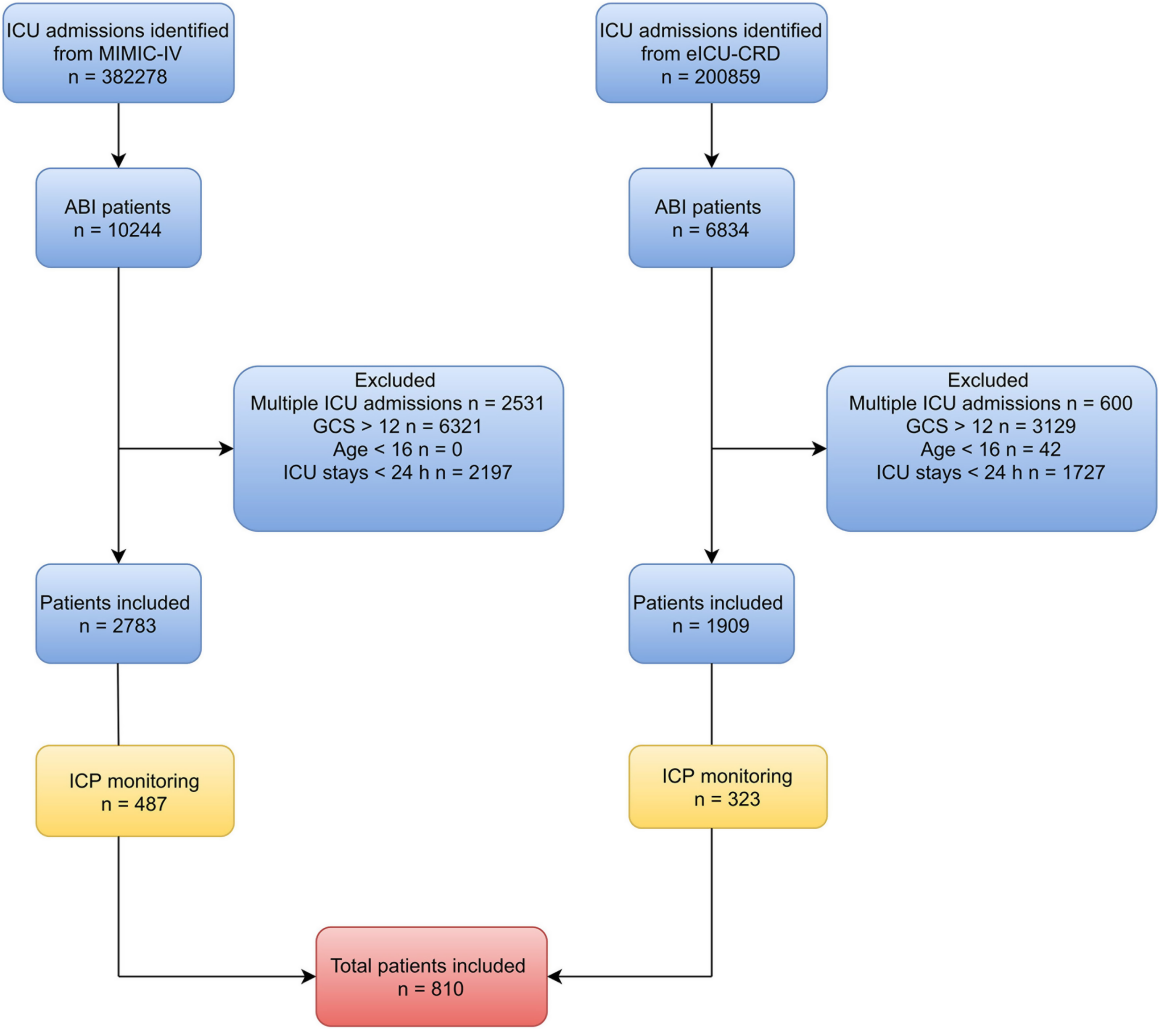
Flowchart of eligible participants

Initially, the search identified 382,278 adult ICU admissions from the MIMIC‐IV database and 200,859 ones from the eICU‐CRD database, respectively. According to the exclusion criteria, 810 patients were finally included in the study cohort (Figure 1). Of these, 600 survived during the whole ICU stay, while the remaining ones did not. Participants in the death‐sample were older [63.00 years (IQR 49.00, 74.00) vs 55.00 years (IQR 39.00, 67.00)], with more comorbidities [4.00 (IQR 2.00, 6.00) vs 3.00 (IQR 1.00, 5.00)], less TBI (32.86% vs 41.83%), more intracranial hemorrhage (40.00% vs 31.67%), more GCS ≤ 8 (93.81% vs 76.00%), higher SOFA [6.00 (IQR 4.00, 8.75) vs 5.00 (IQR 3.00, 7.00)], APSIII [48.00 (IQR 38.00, 70.00) vs 39.00 (IQR 29.00, 55.00)], more mannitol use (31.90% vs 17.33%), higher ICP values [median ICP > 20 mmHg, *n* (%): 12.57% vs 3.61%, median ICP > 22 mmHg, *n* (%): 9.42% vs 2.09%] (Table S2).

### 4.2 Characterization of ICP trajectories

**FIGURE 2:**
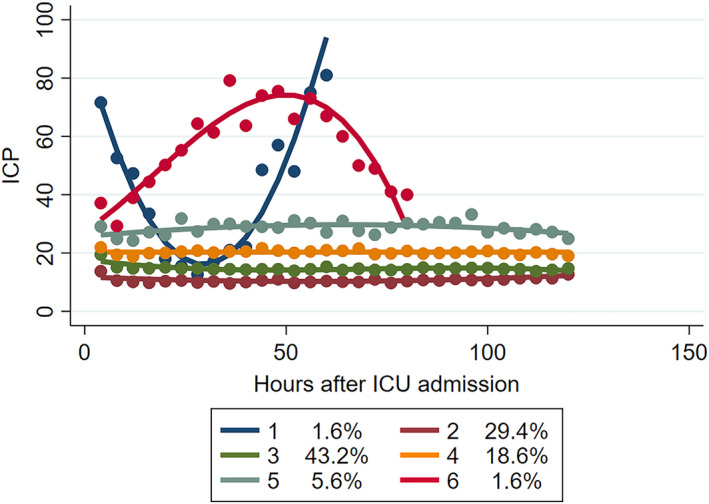
ICP trajectory group characteristics in total cohort

Six distinct trajectory groups were ultimately identified by our model. The AvePP of group membership was ≥0.85 with the smallest BIC, AIC and LL, indicating excellent grouping (Table S3). Longitudinal ICP trajectories were progressively different within each group (Figure 2). Group 1 (*n* = 13), the highest ICP trajectory, was characterized by the first decline then rise over time. Group 2 (*n* = 240) started with the lowest ICP which remained stable over time. Compared with 2, group 3 (*n* = 354), the largest group, had higher ICPs but remained persistently low. It had double‐digit ICPs, but almost no value can exceed 20 mmHg. Group 4 (*n* = 146) had ICP values floating around 20 mmHg. Group 5 (*n* = 45) had elevated ICP‐ fluctuating between 20–30 mmHg. Group 6 (*n* = 12) was a small cohort with early high ICP that rose first continued to decline over the next 90 h. Of 810 subjects included, 1.6% were classified group 1 (“the highest ICP, declined then rose, with a high volatility”), 29.4% group 2 (“the lowest ICP, stable”), 43.2% group 3 (“low ICP, stable”), 18.6% group 4 (“medium ICP, stable”), 5.6% group 5 (“high ICP, intermittent spikes”), 1.6% group 6 (“high, rose then declined, with a high volatility in ICP”).

The demographic and clinical characteristics stratified by the ICP trajectories groups are shown in Table 1 and Table S4. Median age in groups 2: 65.00 years (IQR 53.00, 77.00) and 3: 59.00 years (IQR 45.00, 70.00) was higher than other groups. Sex and race distributions were also significantly different between the six trajectory groups. Compared with higher ICP group (Group 1, 6), a greater proportion of participants in the relatively low ICP trajectory had smoking history (18.33%, 27.97%, 26.03%, 26.67%). In addition, the proportion of patients demonstrating any type of comorbidities was the highest in groups 2 and 3 [4.00 (IQR 2.00, 6.00), 4.00 (IQR 2.00, 5.00)]. Injury types were significantly different between the three trajectory groups. For example, groups 2 and 3 were mainly comprised of subarachnoid hemorrhage (59.17%, 58.19%), whereas TBI primarily occurred in the remaining four groups (53.85%, 59.59%, 66.67%, and 83.33%). Further, scoring systems, including SOFA, APSIII, and ISS were significantly different between the six trajectory groups. For example, the SOFA and APSIII in group 1, 6 were considerably higher than in other groups [SOFA: 8.00 (IQR 7.00, 9.00), 7.00 (5.00, 8.00), APSIII: 69.00 (IQR 61.00, 77.00), 71.00 (IQR 37.00, 92.00)]. Moreover, patients assigned to group 1,6 had higher ICP and lower cerebral perfusion pressure (CPP) than other groups [ICP: 29.00 (IQR 21.00, 47.00), 20.50 (IQR 13.00, 31.00), CPP: 57.00 (IQR 53.00, 62.00), and 65.50 (IQR 55.50, 75.50)]. Additionally, the proportion of subjects who received craniectomy or ventriculostomy was the highest in group 5 and group 6, respectively (13.33%, 33.33%). To further explore the ICP distribution within different groups, the mean ICP, median number and proportion of ICP spikes are listed in the Table S5. These conventionally reported values were quite different between these six groups.

**TABLE 1:** Demographic and clinical characteristics stratified by the ICP trajectory groups

Variables	Group 1 (*n* = 13) the highest, declined then rose	Group 2 (*n* = 240) the lowest, stable	Group 3 (*n* = 354) low, stable	Group 4 (*n* = 146) medium, stable	Group 5 (*n* = 45) high, intermittent spikes	Group 6 (*n* = 12) rose then declined, with a high volatility in ICPs	*p* Value
Demographics							
Median age (IQR), year	48.00 (30.00, 61.00)	65.00 (53.00, 77.00)	59.00 (45.00, 70.00)	45.50 (29.00, 57.00)	39.00 (25.00, 49.00)	36.00 (23.50, 52.00)	<0.001
Male, *n* (%)	7 (53.85)	122 (50.83)	191 (53.95)	102 (69.86)	31 (68.89)	9 (75.00)	0.002
Race, *n* (%)							0.013
Black	0 (0.00)	14 (5.83)	17 (4.80)	6 (4.11)	2 (4.44)	0 (0.00)	
White	6 (46.15)	162 (67.50)	206 (58.19)	88 (60.27)	30 (66.67)	6 (50.00)	
Hispanic	4 (30.77)	7 (2.92)	20 (5.65)	3 (2.05)	1 (2.22)	1 (8.33)	
Asian	0 (0.00)	6 (2.50)	8 (2.26)	4 (2.74)	1 (2.22)	0 (0.00)	
Others	3 (23.08)	51 (21.25)	103 (29.10)	45 (30.82)	11 (24.44)	5 (41.67)	
BMI (IQR), kg/m^2^	28.50 (25.40, 34.95)	25.85 (23.28, 30.50)	27.30 (23.70, 31.20)	27.60 (23.75, 31.40)	27.70 (24.12, 32.05)	24.40 (23.80, 32.70)	0.911
Smoking history	0 (0.00)	44 (18.33)	99 (27.97)	38 (26.03)	12 (26.67)	0 (0.00)	0.008
CCI, median (IQR)	3.00 (0.00, 4.00)	4.00 (2.00, 6.00)	4.00 (2.00, 5.00)	2.00 (0.00, 3.00)	1.00 (0.00, 3.00)	0.00 (0.00, 2.50)	<0.001
Type of injury, *n* (%)							
TBI	7 (53.85)	77 (32.08)	109 (30.79)	87 (59.59)	30 (66.67)	10 (83.33)	<0.001
Subarachnoid hemorrhage	6 (46.15)	142 (59.17)	206 (58.19)	57 (39.04)	19 (42.22)	5 (41.67)	0.001
Intracranial hemorrhage	3 (23.08)	87 (36.25)	130 (36.72)	39 (26.71)	13 (28.89)	2 (16.67)	0.157
Mechanism of injury, *n* (%)							
Unintentional falls	0 (0.00)	4 (1.67)	13 (3.67)	6 (4.11)	4 (8.89)	0 (0.00)	0.178
Motor vehicle crashes	3 (23.08)	48 (20.00)	113 (31.92)	39 (26.71)	17 (37.78)	3 (25.00)	0.025
Firearm injuries	0 (0.00)	0 (0.00)	1 (0.28)	0 (0.00)	1 (2.22)	0 (0.00)	0.148
Scoring systems							
GCS							0.453
9–12	1 (7.69)	48 (20.00)	76 (21.47)	23 (15.75)	6 (13.33)	3 (25.00)	
≤8	12 (92.31)	192 (80.00)	278 (78.53)	123 (84.25)	39 (86.67)	9 (75.00)	
SOFA	8.00 (7.00, 9.00)	5.00 (3.00, 7.00)	5.00 (3.00, 7.00)	6.00 (3.00, 8.00)	6.00 (3.00, 8.00)	7.00 (5.00, 8.00)	<0.001
APSIII	69.00 (61.00, 77.00)	44.00 (35.25, 60.75)	39.50 (31.00, 54.00)	37.50 (25.00, 65.25)	42.00 (27.50, 64.00)	71.00 (37.00, 92.00)	<0.001
ISS							<0.001
≥ 15	2 (16.67)	36 (15.00)	47 (13.31)	45 (30.82)	14 (31.11)	6 (50.00)	
<15	10 (83.33)	204 (85.00)	306 (86.69)	101 (69.18)	31 (68.89)	6 (50.00)	
Medication							
Mannitol use	4 (30.77)	38 (15.83)	53 (14.97)	43 (29.45)	26 (57.78)	7 (58.33)	<0.001
Hypertonic saline use	10 (76.92)	137 (57.08)	159 (44.92)	93 (63.70)	36 (80.00)	10 (83.33)	<0.001
Surgery							
Craniectomy	0 (0.00)	6 (2.50)	16 (4.52)	14 (9.59)	6 (13.33)	1 (8.33)	0.006
Ventriculostomy	2 (15.38)	58 (24.17)	51 (14.41)	27 (18.49)	5 (11.11)	4 (33.33)	0.027
CSF drainage	1 (7.69)	40 (16.67)	36 (10.17)	20 (13.70)	2 (4.44)	2 (16.67)	0.113
ICP monitoring							
Median ICP (IQR), mmHg	29.00 (21.00, 47.00)	6.00 (5.00, 8.75)	10.00 (9.00, 12.00)	13.00 (11.00, 16.00)	18.00 (15.00, 22.00)	20.50 (13.00, 31.00)	<0.001
Median ICP > 20 mmHg (IQR)	37.50 (24.50, 52.25)	27.00 (27.00, 27.00)	22.50 (22.00, 24.50)	22.50 (21.00, 24.00)	23.00 (22.00, 30.00)	26.00 (23.00, 31.00)	0.012
Median ICP > 20 mmHg, *n* (%)	10 (76.92)	1 (0.50)	6 (1.87)	6 (4.58)	15 (37.50)	5 (50.00)	<0.001
Median ICP > 22 mmHg (IQR)	45.00 (31.25, 54.25)	27.00 (27.00, 27.00)	25.00 (24.00, 28.00)	24.00 (24.00, 25.50)	29.00 (24.00, 32.00)	26.00 (23.00, 31.00)	0.077
Median ICP > 22 mmHg, *n* (%)	8 (61.54)	1 (0.50)	3 (0.93)	3 (2.29)	9 (22.50)	5 (50.00)	<0.001
CPP	57.00 (53.00, 62.00)	78.00 (72.00, 85.00)	76.00 (70.00, 82.00)	73.00 (68.25, 80.00)	70.00 (62.00, 75.50)	65.50 (55.50, 75.50)	<0.001

Abbreviation: ICP, intracranial pressure; IQR, interquartile range; BMI, body mass index; CCI, Charlson comorbidity index; TBI, traumatic brain injury; GCS, Glasgow Coma Score; SOFA, sepsis‐related organ failure assessment; APSIII, acute physiology score III; ISS, injury severity score; CSF, cerebrospinal fluid; CPP, cerebral perfusion pressure.

### 4.3 ICP trajectories and clinical outcomes

**FIGURE 3:**
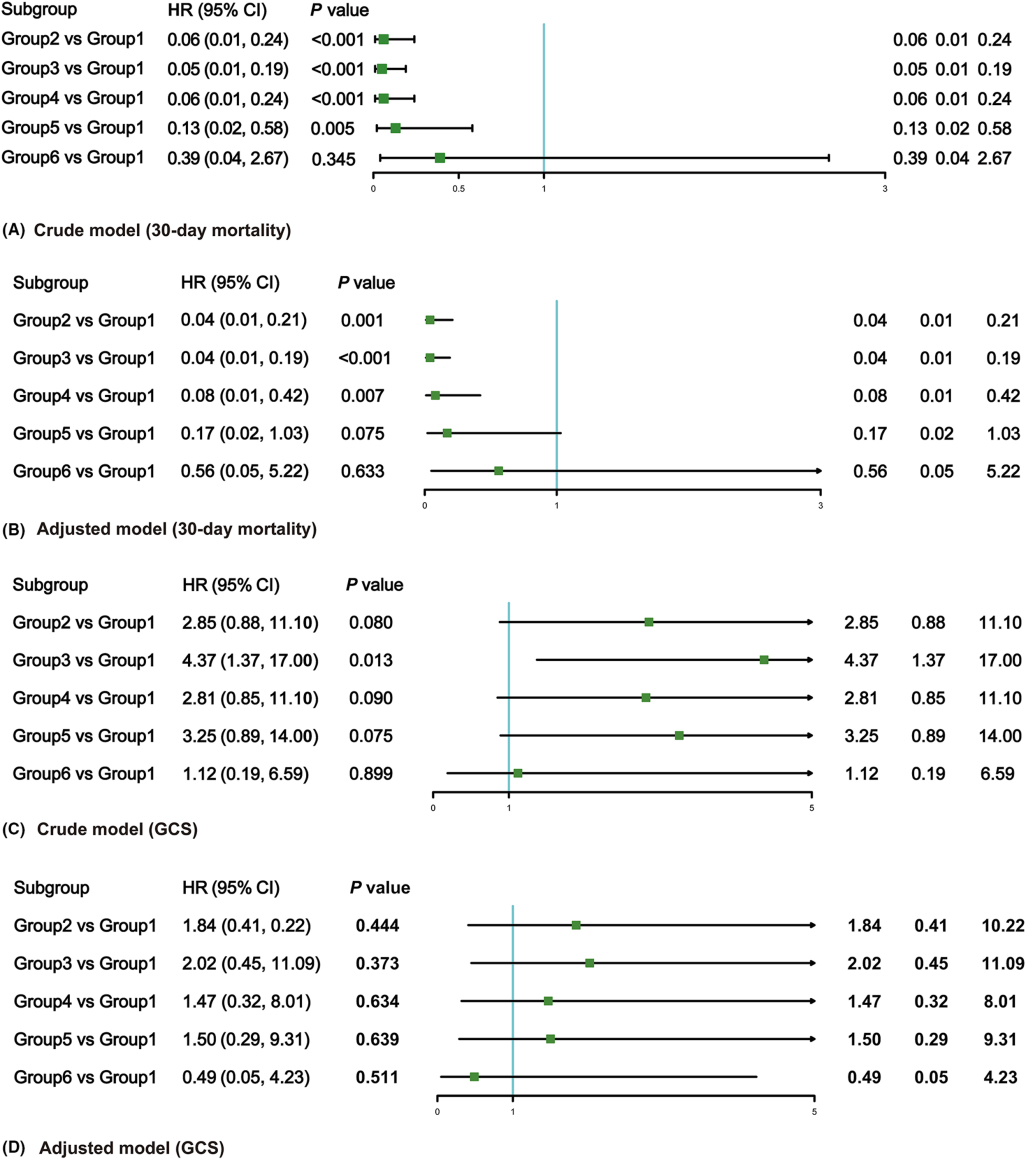
Multinomial logistic regression analysis for the association between ICP trajectories and clinical outcomes

In the unadjusted models, belonging to groups 2, 3, 4, and 5 was associated with reduced mortality (odds ratio [OR] 0.06; 95% confidence interval [CI] 0.01, 0.24), (OR 0.05; 95% CI 0.01, 0.19), (OR 0.06; 95% CI 0.01, 0.24), (OR 0.13; 95% CI 0.02, 0.58) and better neurological outcome (OR 4.37; 95% CI 1.37, 17.0) (group 1 as a reference), suggesting favorable outcomes in these four groups. After adjusting for potential confounders, the multinomial logistic regression model yielded similar results, only with group 5 having no statistically significant results (Mortality: OR 0.17; 95% CI 0.02, 1.03) (Figure 3). To increase ‐ robustness, logistic models to explore the predictors of clinical outcome were performed. In keeping with previous studies, risks factors identified in our paper incorporated average ICP, age, BMI, smoking history, initial GCS, and type of injury. It is worth noting that all these confounders have been properly adjusted in our models (Table S6).

### 4.4 Estimating membership in a given trajectory

On multinomial analysis, as the age increased, the odds of being in group 3, 4, 5, 6 decreased. For example, odds of being in the group 6 (rose then declined, with a high volatility in ICP) vs the group 2 (the lowest, stable) decreased by a factor of (OR 0.91; 95% CI 0.86, 0.97) for each age increase. Also, those treated with medication after ICU admission were more likely to be in the group 5 (OR 15.01; 95% CI 4.87, 46.33) when compared to group 2. Conversely, treatment with surgery also decreased odds of the membership from group 2 to group 5, indicating favorable outcomes (Table S7).

### 4.5 Validation of previous ICP threshold

The results of ROC (30‐day mortality) revealed that the area under the curve (AUC) was only 0.528, accompanied by a low value of sensitivity (0.11) and specificity (0.94) when setting the threshold 22 mmHg as the cutoff value. With regard to GCS value at discharge from ICU, the AUC was 0.523, with a low value of sensitivity (0.95) and specificity (0.10). Similar results were found in elder patients and female patients, suggesting that “one fixed, universal ICP‐treatment‐threshold fits all” approach deserved to be questioned (Table S8). Perhaps, it may be more appropriate to focus on the ICP evolution trajectories than absolute values.

### 4.6 Age and ICP trajectories

**FIGURE 4:**
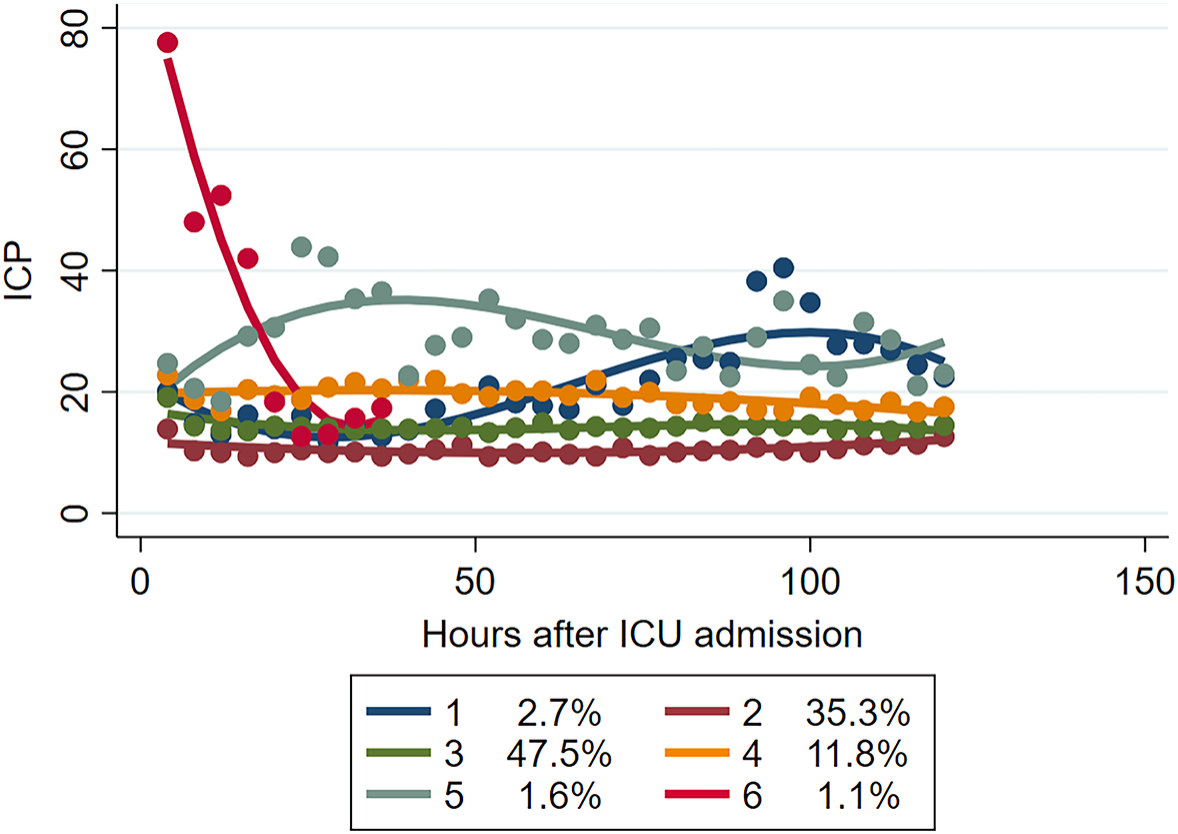
ICP trajectory group characteristics in elderly patients

Should ICP management in older patients be different to younger ones? In order to elucidate the aforementioned issues, we performed GBTM in this specific subject. We found that the GBTM yielded fundamentally different ICP patterns (Figure 4). Compared with the total cohort trajectory, the group 6 (the highest, plunging) had large changes in fluctuations. And the logistic model showed that group 2 (the lowest, stable), 3 (the relatively low, stable) was associated with reduced mortality (OR 0.09; 95% CI 0.01, 0.77), (OR 0.08; 95% CI 0.01, 0.71) when compared to group 6 (Table S9). This ICP trajectory classified subjects into two phenotypes, namely, the favorable group (group 2, 3) and unfavorable group (group 1, 4, 5, 6).

## 5 DISCUSSION

In cerebrovascular disease, high variations in ICPs remain a topic of concern, if unproperly treated, will be accompanied by hydrocephalus.^27^ And researchers have employed machine learning to predict the trans‐stenotic pressure gradient in patients with idiopathic intracranial hypertension while others have used glyceryl trinitrate to decrease ICPs in ischemic stroke patients.^28^, ^29^ And in this paper, we focused on specific patients who are plagued by ABI. In this large cohort of ABI patients, GBTMs revealed six distinct ICP patterns over the ICU hospitalization period, adding nuance to time‐invariant point values. Three groups of patients displayed unfavorable results while the other three groups reported favorable outcomes. Another important finding worth noting was that the ICP trajectory was fundamentally different across all elderly patients compared to the total cohorts. Interestingly, our study demonstrated that in our dataset, the previous threshold did not indicate a good predictive performance. Elevated ICP is harmful as it may hinder the delivery of adequate nutrients to the brain. In the extreme cases, ICP elevation exceeds arterial pressure and then prevents intracranial blood flow, as is seen with brain death.^11^

Indeed, this study identified six distinct ICP trajectory groups which were distinguished by different ICP patterns, and in the risk‐adjusted models, these trajectories were significantly associated with clinical outcomes, such as 30‐day mortality. Groups 2, 3, and 4 had stable trajectories with a value of ICP less than 20 mmHg, and naturally, reported a favorable outcome. Nonetheless, in a research conducted by Jha RM et al, two cohorts of patients had unfavorable outcomes despite low ICP.^24^ Authors tried to explain the association by differences in cerebral compliance, neuroplasticity, neurovascular‐coupling, but finally failed, and they concluded that this result was beyond the scope of their manuscript. Intracranial hypertension (defined as ICP values of greater than 20–25 mmHg) is considered pathologically significant due to its correlation to mortality,^30^ this finding was further confirmed in our study. Groups 1, 5, and 6 had high ICP (greater than 20 mmHg), of these, high variation was found in group 1 and group 6. These three groups reported increased odds of unfavorable outcomes. In clinical practice, if the patient has such an ICP evolution pattern, relevant measures should be taken as soon as possible. We hypothesized that ICP “variability” may be an important factor associated with clinical outcomes. Responsive elastance, or neuroplasticity, could be a potential explanation that warrants further exploration. A clinical study conducted by Robba et al^9^ also found that use of ICP monitoring may be associated with better neurological outcome and lower 6‐month mortality in more severe cases, which further illustrates the importance of early attention of ICPs. Interestingly, our study demonstrated that medication was associated with unfavorable outcomes. The underlying assumptions for those analyses were the inherent nature of observation study, that is, the cause and effect were confused. People in these two groups tended to be in the queue with bad condition, as evidenced by the baseline variables. Moreover, the confidence intervals for the findings were wide, reducing the power of the study to detect a clinically important difference.

Taken together, in the last four editions of the BTF Guidelines for the management of TBI, the recommended ICP threshold for treatment has changed from 25 mmHg^31^ to 20 mmHg ^32^ to 22 mmHg.^7^ No defined consensus is available to guide clinicians in hemorrhagic stroke (intracerebral hemorrhage and subarachnoid hemorrhage), the experience for ICP monitoring in these patients is mostly from TBI. Further, the new generic threshold of 22 mmHg proposed in the new BTF guideline, was quite questionable, because it stemmed from one single‐center retrospective observation study. The study incorporated 459 patients admitted with TBI to the Addenbrooke's hospital, Cambridge, UK and identified threshold values for ICP based on sequential chi‐square tests.^15^ Also, Sorrentino et al included patients who underwent decompressive craniectomy yet without an effective adjustment for this confounder, which may impact this described population‐based threshold.^33^ Finally, multiple phenotypes with differing pathophysiology of raised ICP as well as the heterogeneity of included patients accelerate its inaccuracy. Note that in our study, the validation of the previous threshold did not demonstrate a very good performance, which further adds evidence that a fixed, universal ICP‐treatment‐threshold is indeed at issue. Specifically, the association between elevated ICP and outcome is not merely attributable to crossing a threshold, but depends upon the dynamic changes in intracranial hypertension. Chesnut RM suggested a novel “traffic light” pattern, wherein a red light (abnormal ICP waveform) indicated that alteration of ICP thresholds was contraindicated, a yellow light (±abnormal ICP waveform) suggested that one should proceed with caution, and a green light (normal ICP waveform) allowed that such a move would be appropriate.^34^ Likewise, Güiza et al^35^ visualized the pressure and time burden of intracranial hypertension and found that ICP above 20 mmHg lasting longer than 37 min was associated with worse outcomes. But in another Belgium cohort, the pressure‐time burden was ICP ≥20 mmHg lasting longer than 13 min.^36^ Detailed data of literature review are listed in Table S10.

When subgroup for age was analyzed, the threshold did not change for mortality but decreased to 18 mmHg for favorable outcome for patients over 55 years of age. Given that subgroup analysis may have been underpowered, and these findings were limited to only this study, the BTF did not support an ICP recommendation that varies by age. In the total model, we found that patients in groups 2 and 3 were older, with higher CCI, more subarachnoid hemorrhage, lower ICP, higher CPP. This may be partially attributed to the special pathophysiological condition of the elderly, including the increase in age‐related comorbidities, use of preinjury antiplatelet/anticoagulants, age‐related atrophy.^11^, ^15^ Accordingly, subgroup analysis was done in elderly patients, producing a totally different ICP trajectory. Cerebral atrophy and increased CSF space could buffer new intracranial hypertension, which could be linked to a lower volatility of ICP in the trajectory.^37^, ^38^ But lower ICPs do not denote a good prognosis, the associated comorbidities, drug induced coagulopathy, lower compliance, diminished brain reserve and reduced neuroplasticity may hamper the clinical outcomes of the elderly.^11^, ^39^ This phenomenon has also been verified in our article. Group 4, a medium, relatively stable ICP group (rare spikes greater than 20 mmHg), had unfavorable outcomes while in the total cohort, the corresponding group 4 boasted favorable outcomes. Thus, there is a pressing need to develop optimum care management including prompt CT scans, systematic physiology monitoring for these patients.

The present study attempted to add to the current knowledge in developing a new concept of “time‐varying” for ICP. In particular, the application of GBTMs in ABI patients enabled the identification of specific ICP patterns over time and the clinical outcomes associated with distinct trajectories. The “dynamic and individualized” concept is replacing ICP regulation with ABI management, with ICP value regarded as a tool, not a goal. Future study could focus on the individualization of ICP and investigate its conjunction with other parameters including the state of cerebrovascular pressure reactivity, tissue oxygenation quality, and non‐ICP‐related metabolic and energy crisis. Furthermore, this study was based on a population‐based longitudinal cohort from multi‐centers in the United States, a high‐quality data with granular temporal detail, a homogeneous population, accordingly, ensuring the robustness, reliability, generalizability of the findings.

This study had several limitations, consistent with those inherent to many large administrative database studies. First, based on electronic records of routine clinical practice, missing data and outliers were common. Apart from this, the use of GCS value at discharge from ICU was suboptimal but necessary because of the lack of data concerning Glasgow Outcome Scale (GOS) at 6 months. Third, variables were extracted by ICD‐9 and ICD‐10 diagnosis codes. Incorrect codes or misclassification bias inevitably exist. Fourthly, the time span of this study is relatively long, the ICP monitoring catheter and treatment may vary in decades. Finally, neuroimaging data including CT and cerebral angiography were not included in the databases.

## 6 CONCLUSIONS

To summarize, we proposed a novel ICP trajectory that could enable us to move treatment of ABI from a fixed threshold approach to a more individualized treatment. ICP values and variability differed across these six identified trajectory groups with favorable vs. unfavorable outcomes. The epidemiological shift toward a larger proportion of physiologically fragile elderly patients calls for more attention. If validated rigorously, ICP trajectory modeling combined with higher resolution data including waveform analysis may provide additional potentially useful classifications for ICP.

## AUTHOR CONTRIBUTIONS

The conception and design was made by Fan Yang, Chi Peng, Liwei Peng, and Peng Wang. Fan Yang and Chi Peng contributed to data acquisition and statistical analysis. : Chi Peng, Peng Wang, and Lei Zhao contributed to analysis and interpretation of data. Wei Zuo and Chao Cheng critically revised the article. Zhichao Jin and Weixin Li contributed to study supervision. All authors reviewed the submitted version of the manuscript.

## CONFLICT OF INTEREST

The authors declare that they have no competing interests.

## Supporting information


Appendix S1

## Data Availability

Availability of data and material: The data used for this study can be accessed via the MIMIC‐IV database (https://mimic.physionet.org/), and eICU database (eICU Collaborative Research Database v2.0 (physionet.org): https://physionet.org/content/eicu‐crd/2.0/); full instructions for obtaining access can be found on the website.
