# Supplementary material for: Group‐based trajectory modeling of intracranial pressure in patients with acute brain injury: Results from multi‐center ICUs, 2008–2019
Source: CNS Neurosci Ther. 2022 May 25;28(8):1218–28. doi: 10.1111/cns.13854 (PMC9253780; doi:10.1111/cns.13854)
Supplement: Supplementary file 1 — Appendix S1 [file CNS-28-1218-s001.docx]

| Table S1 Missing number (%) for included variables in the dataset | |
| --- | --- |
| Variables | Missing, N (%) |
| BMI | 15.04 |
| ISS | 0.25 |
| APSIII | 2.47 |
| Median ICP | 11.47 |
| CPP | 28.24 |
| GCS value at  discharge from ICU | 0.25 |
| Abbreviations: BMI = body mass index; ISS = injury severity score | |
| APSIII = acute physiology score III; ICP = intracranial pressure; | |
| CPP = cerebral perfusion pressure; GCS = Glasgow Coma Score; | |
| ICU = intensive care unit |  |

| Table S2 Baseline characteristic of the included  ABI patients | | | | |  |
| --- | --- | --- | --- | --- | --- |
| Variables | Total (n = 810) | Survival (n = 600) | Death (n = 210) | *p* Value |  |
| **Demographics** |  |  |  |  |  |
| Median age (IQR), y | 57.00 (42.00, 69.00) | 55.00 (39.00, 67.00) | 63.00 (49.00, 74.00) | < 0.001 |  |
| Male, n (%) | 462 (57.04) | 348 (58.00) | 114 (54.29) | 0.393 |  |
| Race, n (%) |  |  |  |  |  |
| Black | 39 (4.81) | 26 (4.33) | 13 (6.19) |  |  |
| White | 498 (61.48) | 380 (63.33) | 118 (56.19) |  |  |
| Hispanic | 36 (4.44) | 27 (4.50) | 9 (4.29) |  |  |
| Asian | 19 (2.35) | 15 (2.50) | 4 (1.90) |  |  |
| Others | 218 (26.91) | 152 (25.33) | 66 (31.43) |  |  |
| BMI (IQR), kg/m^2^ | 26.70 (23.67, 31.13) | 26.60 (23.50, 30.70) | 27.50 (24.33, 31.87) | 0.106 |  |
| Smoking history | 193 (23.83) | 145 (24.17) | 48 (22.86) | 0.772 |  |
| Coexisting disorders, n (%) |  |  |  |  |  |
| Myocardial infarction | 28 (3.46) | 21 (3.50) | 7 (3.33) | 1.000 |  |
| Congestive heart failure | 40 (4.94) | 24 (4.00) | 16 (7.62) | 0.058 |  |
| Peripheral vascular disease | 35 (4.32) | 30 (5.00) | 5 (2.38) | 0.159 |  |
| Cerebrovascular disease | 420 (51.85) | 307 (51.17) | 113 (53.81) | 0.562 |  |
| Dementia | 9 (1.11) | 4 (0.67) | 5 (2.38) | 0.055 |  |
| chronic pulmonary disease: | 70 (8.64) | 46 (7.67) | 24 (11.43) | 0.127 |  |
| Rheumatic disease | 4 (0.49) | 2 (0.33) | 2 (0.95) | 0.277 |  |
| Peptic ulcer disease | 7 (0.86) | 7 (1.17) | 0 (0.00) | 0.200 |  |
| Diabetes | 114 (14.07) | 74 (12.33) | 40 (19.05) | 0.022 |  |
| Paraplegia | 83 (10.25) | 61 (10.17) | 22 (10.48) | 1.000 |  |
| Renal disease | 28 (3.46) | 12 (2.00) | 16 (7.62) | < 0.001 |  |
| Malignant cancer | 17 (2.10) | 12 (2.00) | 5 (2.38) | 0.781 |  |
| Severe liver disease | 10 (1.23) | 4 (0.67) | 6 (2.86) | 0.023 |  |
| Metastatic solid tumor | 10 (1.23) | 5 (0.83) | 5 (2.38) | 0.137 |  |
| AIDS | 1 (0.12) | 1 (0.17) | 0 (0.00) | 1.000 |  |
| CCI, median (IQR) | 3.00 (1.00, 5.00) | 3.00 (1.00, 5.00) | 4.00 (2.00, 6.00) | < 0.001 |  |
| **Type of injury, n (%)** |  |  |  |  |  |
| TBI | 320 (39.51) | 251 (41.83) | 69 (32.86) | 0.027 |  |
| Subarachnoid hemorrhage | 435 (53.70) | 329 (54.83) | 106 (50.48) | 0.313 |  |
| Intracranial hemorrhage | 274 (33.83) | 190 (31.67) | 84 (40.00) | 0.035 |  |
| **Mechanism of injury, n (%)** |  |  |  |  |  |
| Unintentional falls | 27 (3.33) | 22 (3.67) | 5 (2.38) | 0.503 |  |
| Motor vehicle crashes | 223 (27.53) | 159 (26.50) | 64 (30.48) | 0.307 |  |
| Firearm injuries | 2 (0.25) | 2 (0.33) | 0 (0.00) | 1.000 |  |
| **Scoring systems** |  |  |  |  |  |
| GCS |  |  |  | < 0.001 |  |
| 9-12 | 157 (19.38) | 144 (24.00) | 13 (6.19) |  |  |
| <= 8 | 653 (80.62) | 456 (76.00) | 197 (93.81) |  |  |
| SOFA | 5.00 (3.00, 7.00) | 5.00 (3.00, 7.00) | 6.00 (4.00, 8.75) | < 0.001 |  |
| APSIII | 41.50 (30.25, 59.00) | 39.00 (29.00, 55.00) | 48.00 (38.00, 70.00) | < 0.001 |  |
| ISS |  |  |  | 0.086 |  |
| >= 15 | 150 (18.56) | 120 (20.03) | 30 (14.35) |  |  |
| < 15 | 658 (81.44) | 479 (79.97) | 179 (85.65) |  |  |
| **Medication** |  |  |  |  |  |
| Mannitol use | 171 (21.11） | 104 (17.33） | 67 (31.90） | <0.001 |  |
| Hypertonic saline use | 255 (31.38) | 182 (30.33） | 73 (34.76） | 0.234 |  |
| **Surgery** |  |  |  |  |  |
| Craniectomy | 43 (5.31) | 33 (5.50) | 10 (4.76) | 0.817 |  |
| Ventriculostomy | 147 (18.15) | 110 (18.33) | 37 (17.62) | 0.899 |  |
| CSF drainage | 101 (12.47) | 79 (13.17) | 22 (10.48) | 0.371 |  |
| **ICP monitoring** |  |  |  |  |  |
| Median ICP (IQR), mmHg | 10.00 (7.00, 13.00) | 10.00 (7.00, 13.00) | 10.00 (8.00, 14.50) | 0.146 |  |
| Median ICP> 20 mmHg (IQR) | 24.00 (22.00, 31.50) | 23.00 (22.00, 26.50) | 28.00 (22.75, 37.00) | 0.090 |  |
| Median ICP> 20 mmHg, n (%) | 43 (6.00) | 19 (3.61) | 24 (12.57) | < 0.001 |  |
| Median ICP> 22 mmHg (IQR) | 29.00 (24.00, 39.00) | 26.00 (24.00, 31.00) | 31.50 (26.25, 46.00) | 0.125 |  |
| Median ICP> 22 mmHg, n (%) | 29 (4.04) | 11 (2.09) | 18 (9.42) | < 0.001 |  |
| CPP | 76.00 (69.00, 82.00) | 76.00 (69.25, 82.00) | 75.00 (67.00, 80.00) | 0.066 |  |
| Abbreviations: ABI = acute brain injury; IQR = interquartile range; BMI = body mass index; AIDS = acquired immunodeficiency syndrome; | | | | |  |
| CCI = Charlson comorbidity index; TBI = traumatic brain injury; GCS = Glasgow Coma Score; SOFA = sepsis related organ failure assessment; | | | | |  |
| APSIII = acute physiology score III; ISS = injury severity score; CSF = cerebrospinal fluid; ICP = intracranial pressure; | | | | |  |
| CPP = cerebral perfusion pressure. |  |  |  |  |  |

| Table S3 The selection process of GBTM | | | | | | |
| --- | --- | --- | --- | --- | --- | --- |
| ICP trajectories | Polynomial order | Avepp | Occ | BIC | AIC | LL |
| 2-group solution | 3 1 | 0.99/0.97 | 15.63/129.40 | -64326 | -64308 | -64300 |
| 3-group solution | 3 0 2 | 0.97/0.93/0.99 | 14.81/41.62/1884.32 | -63487 | -63461 | -63450 |
| 4-group solution | 3 1 0 3 | 0.95/0.91/0.94/0/95 | 11.68/21.37/185.26/759.80 | -63062 | -63027 | -63012 |
| 5-group solution | 3 3 3 1 2 | 0.96/0.95/0.90/0.94/0.95 | 1608.23/13.47/20.52/178.81/1311.30 | -62788 | -62736 | -62714 |
| 6-group solution | 2 2 3 0 2 3 | 0.98/0.87/0.86/0.89/0.92/0.98 | 2822.97/15.61/8.21/37.61/197.81/3779.48 | -62614 | -62558 | -62534 |

Abbreviations: GBTM = group-based trajectory modeling; ICP = intracranial pressure; AvePP = average posterior probability; Occ = odds of correct classification; BIC = Bayesian information criterion; AIC = Akaike information criterion; LL = Log-likelihood.

| Table S4 Coexisting disorders of the included ABI patients | | | | | | | |
| --- | --- | --- | --- | --- | --- | --- | --- |
| Coexisting disorders, n (%) | Group 1 (n = 13) high, declined then rose | Group 2 (n = 240) low, stable | Group 3 (n = 354) intermittent spikes | Group 4 (n = 146) medium, stable | Group 5 (n = 45) consistently high | Group 6 (n = 12) high, rose then declined | *p* Value |
| Myocardial infarction | 1 (7.69) | 9 (3.75) | 14 (3.95) | 3 (2.05) | 1 (2.22) | 0 (0.00) | 0.774 |
| Congestive heart failure | 0 (0.00) | 16 (6.67) | 19 (5.37) | 5 (3.42) | 0 (0.00) | 0 (0.00) | 0.306 |
| Peripheral vascular disease | 1 (7.69) | 9 (3.75) | 17 (4.80) | 7 (4.79) | 1 (2.22) | 0 (0.00) | 0.870 |
| Cerebrovascular disease | 3 (23.08) | 119 (49.58) | 229 (64.69) | 53 (36.30) | 15 (33.33) | 1 (8.33) | < 0.001 |
| Dementia | 0 (0.00) | 6 (2.50) | 3 (0.85) | 0 (0.00) | 0 (0.00) | 0 (0.00) | 0.232 |
| Chronic pulmonary disease | 1 (7.69) | 25 (10.42) | 32 (9.04) | 11 (7.53) | 1 (2.22) | 0 (0.00) | 0.447 |
| Rheumatic disease | 0 (0.00) | 2 (0.83) | 2 (0.56) | 0 (0.00) | 0 (0.00) | 0 (0.00) | 0.893 |
| Peptic ulcer disease | 0 (0.00) | 1 (0.42) | 5 (1.41) | 0 (0.00) | 1 (2.22) | 0 (0.00) | 0.513 |
| Diabetes | 1 (7.69) | 41 (17.08) | 48 (13.56) | 21 (14.38) | 2 (4.44) | 1 (8.33) | 0.297 |
| Paraplegia | 0 (0.00) | 22 (9.17) | 41 (11.58) | 19 (13.01) | 0 (0.00) | 1 (8.33) | 0.115 |
| Renal disease | 0 (0.00) | 8 (3.33) | 14 (3.95) | 4 (2.74) | 2 (4.44) | 0 (0.00) | 0.910 |
| Malignant cancer | 0 (0.00) | 4 (1.67) | 11 (3.11) | 2 (1.37) | 0 (0.00) | 0 (0.00) | 0.572 |
| Severe liver disease | 1 (7.69) | 4 (1.67) | 3 (0.85) | 2 (1.37) | 0 (0.00) | 0 (0.00) | 0.309 |
| Metastatic solid tumor | 0 (0.00) | 2 (0.83) | 6 (1.69) | 1 (0.68) | 0 (0.00) | 1 (8.33) | 0.223 |
| AIDS | 0 (0.00) | 1 (0.42) | 0 (0.00) | 0 (0.00) | 0 (0.00) | 0 (0.00) | 0.795 |
| Abbreviations: ABI = acute brain injury; AIDS = acquired immunodeficiency syndrome. | | | | |  |  |  |

| Table S5A-E: ICP differences Between Trajectory Groups | | | | | |
| --- | --- | --- | --- | --- | --- |
| A: Average ICP is different between ICP-trajectory groups | | | | | |
| ICP Trajectory Group  (Average ICP) | 1 (34.1 mmHg) | 2 (8.3 mmHg) | 3 (10.9 mmHg) | 4 (14.2 mmHg) | 5 (18.8 mmHg) |
| 2 (8.3 mmHg) | <0.001 |  |  |  |  |
| 3 (10.9 mmHg) | <0.001 | <0.001 |  |  |  |
| 4 (14.2 mmHg) | 0.063 | <0.001 | <0.001 |  |  |
| 5 (18.8 mmHg) | 1.000 | <0.001 | <0.001 | 0.246 |  |
| 6 (25.4 mmHg) | 1.000 | <0.001 | 0.001 | 1.000 | 1.000 |

| B: Median number of ICP spikes > 20 mmHg is different between ICP trajectory groups | | | | | |
| --- | --- | --- | --- | --- | --- |
| ICP Trajectory Group  (Median ICP > 20mmHg) | 1 (10) | 2 (21) | 3 (18) | 4 (15) | 5 (17) |
| 2 (21) | <0.001 |  |  |  |  |
| 3 (18) | <0.001 | 1.000 |  |  |  |
| 4 (15) | <0.001 | 1.000 | 1.000 |  |  |
| 5 (17) | 0.001 | <0.001 | <0.001 | <0.001 |  |
| 6 (7) | 1.000 | <0.001 | <0.001 | <0.001 | 0.639 |

| C: Proportion of ICP spikes > 20 mmHg is different between ICP trajectory groups | | | | | |
| --- | --- | --- | --- | --- | --- |
| ICP Trajectory Group  (% ICP > 20mmHg) | 1 (76.92%) | 2 (0.50%) | 3 (1.87%) | 4 (4.58%) | 5 (37.50%) |
| 2 (0.50%) | <0.001 |  |  |  |  |
| 3 (1.87%) | <0.001 | 1.000 |  |  |  |
| 4 (4.58%) | <0.001 | 1.000 | 1.000 |  |  |
| 5 (37.50%) | 1.000 | <0.001 | <0.001 | <0.001 |  |
| 6 (50%) | 1.000 | <0.001 | <0.001 | <0.001 | 1.000 |

| D: Median number of ICP spikes > 22 mmHg is different between ICP trajectory groups | | | | | |
| --- | --- | --- | --- | --- | --- |
| ICP Trajectory Group  (Median ICP > 22mmHg) | 1 (8) | 2 (4) | 3 (8) | 4 (10) | 5 (10) |
| 2 (4) | <0.001 |  |  |  |  |
| 3 (8) | <0.001 | 1.000 |  |  |  |
| 4 (10) | <0.001 | 0.518 | 0.672 |  |  |
| 5 (10) | <0.001 | <0.001 | <0.001 | 0.002 |  |
| 6 (6) | 1.000 | <0.001 | <0.001 | <0.001 | 0.003 |

| E: Proportion of ICP spikes > 22 mmHg is different between ICP trajectory groups | | | | | |
| --- | --- | --- | --- | --- | --- |
| ICP Trajectory Group  (% ICP > 22mmHg) | 1 (61.54%) | 2 (1.69%) | 3 (2.27%) | 4 (6.85%) | 5 (22.22%) |
| 2 (1.69%) | <0.001 |  |  |  |  |
| 3 (2.27%) | <0.001 | 1.000 |  |  |  |
| 4 (6.85%) | <0.001 | 1.000 | 1.000 |  |  |
| 5 (22.22%) | 1.000 | <0.001 | <0.001 | <0.001 |  |
| 6 (50%) | 1.000 | <0.001 | <0.001 | <0.001 | 1.000 |
| Abbreviations: ICP = intracranial pressure | | | | | |

| Table S6 Logistic regression analyses of traditional multivariable models  predicting clinical outcomes | | | | |
| --- | --- | --- | --- | --- |
| Variables | Mortality | | GCS | |
|  | OR (95% CI) | *P* Value | OR (95% CI) | *P* Value |
| Average ICP | 1.05 (1.02, 1.08) | 0.001 | 0.97 (0.95-1.01)) | 0.059 |
| Age | 1.02 (1.01, 1.04) | 0.002 | 0.99 (0.96-1.01) | 0.055 |
| Sex | 0.95 (0.64, 1.39) | 0.782 | 1.13 (0.81-1.59) | 0.478 |
| BMI | 1.03 (1.01, 1.06) | 0.015 | 0.99 (0.97-1.02) | 0.823 |
| Smoking history | 1.05 (0.67, 1.64) | 0.798 | 1.88 (1.26-2.85) | 0.002 |
| CCI | 1.09 (0.97, 1.22) | 0.126 | 1.06 (0.95-1.19) | 0.288 |
| Initial GCS | 1.20 (1.12, 2.56) | 0.011 | 0.31 (0.18-0.49) | < 0.001 |
| **Type of injury, n (%)** |  |  |  |  |
| TBI | 0.42 (0.21, 0.81) | 0.011 | 0.44 (0.25-0.75) | 0.003 |
| Subarachnoid hemorrhage | 0.60 (0.37, 0.95) | 0.033 | 0.71 (0.48-1.06) | 0.095 |
| Intracranial hemorrhage | 1.02 (0.64, 1.62) | 0.908 | 0.77 (0.52-1.14) | 0.200 |
| SOFA | 1.06 (0.97, 1.15) | 0.148 | 1.06 (0.99-1.14) | 0.093 |
| APSIII | 1.02 (1.01, 1.03) | 0.001 | 0.97 (0.96-0.98) | < 0.001 |
| ISS | 1.01 (0.98, 1.04) | 0.535 | 1.01 (0.98-1.03) | 0.636 |
| Abbreviations: GCS = Glasgow Coma Score; OR = odds ratio; CI= confidence interval; | | | | |
| ICP = intracranial pressure; BMI = body mass index; CCI = Charlson comorbidity index; | | | | |
| TBI = traumatic brain injury; SOFA = sepsis related organ failure assessment; | | | | |
| APSIII = acute physiology score III; ISS = injury severity score | | | | |

| Table S7 Results of multinomial regression analysis examining predictors of GBTM, using group 2 as the reference group | | | | | | | | | | |
| --- | --- | --- | --- | --- | --- | --- | --- | --- | --- | --- |
| Variables | Group 1 vs group 2 | | Group 3 vs Group2 | | Group 4 vs Group 2 | | Group 5 vs Group 2 | | Group 6 vs Group 2 | |
|  | OR (95% CI) | *P* Value | OR (95% CI) | *P* Value | OR (95% CI) | *P* Value | OR (95% CI) | *P* Value | OR (95% CI) | *P* Value |
| Age | 0.97 (0.91, 1.04) | 0.397 | 0.96 (0.94, 0.98) | < 0.001 | 0.93 (0.91, 0.96) | < 0.001 | 0.95 (0.91, 0.99) | 0.015 | 0.91 (0.86, 0.97) | 0.002 |
| Sex | 0.34 (0.09, 1.38) | 0.131 | 0.94 (0.64, 1.38) | 0.761 | 1.28 (0.76, 2.15) | 0.353 | 1.24 (0.47, 3.26) | 0.666 | 1.90 (0.36, 9.92) | 0.447 |
| BMI | 1.07 (0.98, 1.16) | 0.128 | 1.01 (0.98, 1.04) | 0.490 | 1.03 (0.99, 1.06) | 0.198 | 1.05 (0.99, 1.12) | 0.130 | 1.01 (0.90, 1.13) | 0.858 |
| CCI | 0.84 (0.46, 1.53) | 0.569 | 1.10 (0.97, 1.25) | 0.140 | 1.10 (0.92, 1.31) | 0.279 | 0.77 (0.51, 1.18) | 0.232 | 1.44 (0.92, 2.24) | 0.111 |
| Initial GCS | 1.67 (0.20, 14.24) | 0.640 | 0.90 (0.56, 1.44) | 0.659 | 0.99 (0.52, 1.90) | 0.980 | 0.75 (0.24, 2.39) | 0.628 | 0.51 (0.09, 2.81) | 0.442 |
| **Type of injury, n (%)** |  |  |  |  |  |  |  |  |  |  |
| TBI | 1.61 (0.26, 10.14) | 0.613 | 0.81 (0.49, 1.33) | 0.395 | 1.11 (0.57, 2.16) | 0.771 | 2.18 (0.66, 7.15) | 0.201 | 5.78 (0.80, 41.87) | 0.082 |
| Subarachnoid hemorrhage | 0.80 (0.16, 4.05) | 0.786 | 0.99 (0.63, 1.60) | 0.997 | 0.54 (0.30, 0.97) | 0.040 | 0.86 (0.34, 2.18) | 0.753 | 1.38 (0.34, 5.64) | 0.657 |
| Intracranial hemorrhage | 0.68 (0.12, 3.81) | 0.664 | 1.01 (0.63, 1.60) | 0.969 | 0.97 (0.53, 1.77) | 0.908 | 1.19 (0.46, 3.11) | 0.717 | 0.73 (0.13, 3.99) | 0.712 |
| Medication | 1.42 (0.37, 5.50) | 0.605 | 1.01 (0.69, 1.50) | 0.934 | 1.52 (0.93, 2.50) | 0.094 | 15.01 (4.87, 46.33) | < 0.001 | 3.00 (0.80, 11.29) | 0.104 |
| Surgery | 0.42 (0.08, 2.21) | 0.307 | 0.63 (0.40, 0.98) | 0.042 | 0.95 (0.54, 1.66) | 0.845 | 0.49 (0.19, 1.24) | 0.132 | 1.18 (0.29, 4.73) | 0.821 |

Abbreviations: GBTM=group, based trajectory model; OR = odds ratio; CI= confidence interval; BMI = body mass index; CCI = Charlson comorbidity index; GCS = Glasgow Coma Score; TBI = traumatic brain injury; SOFA = sepsis related organ failure assessment; APSIII = acute physiology score III; ISS = injury severity score

| Table S8 Validation of the ICP threshold in the guidelines | | | |
| --- | --- | --- | --- |
| A Validation of the ICP threshold in the guidelines (30-day mortality) | | | |
|  | 22mmHg (Total cohorts) | 18mmHg (Elderly patients) | 18mmHg (Female patients) |
| AUC | 0.528 | 0.500 | 0.507 |
| Sensitivity | 0.11 | 0.00 | 0.91 |
| Specificity | 0.94 | 100.00 | 0.10 |
| PPV | 0.57 | 0.50 | 0.77 |
| NPV | 0.76 | 0.71 | 0.76 |
| LR | 4.04 | 2.40 | 9.32 |
| Abbreviations: ICP = intracranial pressure; AUC = area under the curve; PPV = positive predictive value | | | |
| NPV = negative predictive value; LR = likelihood ratio. | | | |
|  |  |  |  |
| B Validation of the ICP threshold in the guidelines (GCS value at discharge from ICU) | | | |
|  | 22mmHg (Total cohorts) | 18mmHg (Elderly patients) | 18mmHg (Female patients) |
| AUC | 0.523 | 0.506 | 0.502 |
| Sensitivity | 0.95 | 0.13 | 0.90 |
| Specificity | 0.10 | 0.88 | 0.10 |
| PPV | 0.61 | 0.61 | 0.60 |
| NPV | 0.63 | 0.50 | 0.45 |
| LR | 1.04 | 1.02 | 1.02 |
| Abbreviations: ICP = intracranial pressure; GCS = Glasgow coma score; ICU = intensive care unit | | | |
| AUC = area under the curve; PPV = positive predictive value; NPV = negative predictive value; | | | |
| LR = likelihood ratio. | | | |

| Table S9 Multinomial logistic regression analysis for the association between ICP trajectories and clinical outcomes in elder patients | | | | | | |
| --- | --- | --- | --- | --- | --- | --- |
| A: Association between ICP trajectories and clinical outcomes in elder patients (crude model) | | | | | | |
| Outcomes | Group 1 | Group 2 | Group 3 | Group 4 | Group 5 | Group 6 |
| **Primary outcomes** |  |  |  |  |  |  |
| All-cause mortality, OR (95% CI) | 0.29 (0.01, 3.15) | 0.12 (0.00, 0.87) | 0.10 (0.00, 0.73) | 0.10 (0.00, 0.79) | 1.45 (0.03, 67.6) | ref |
| P Value | 0.336 | 0.035 | 0.022 | 0.833 | 0.833 |  |
| GCS value at  discharge from ICU, OR (95% CI) | 3.14 (0.32, 39.9) | 1.62 (0.24, 14.2) | 3.17 (0.47, 27.8) | 1.77 (0.25, 16.3) | 1.86 (0.17, 25.4) | ref |
| P Value | 0.334 | 0.617 | 0.228 | 0.565 | 0.621 |  |
|  |  |  |  |  |  |  |
|  | | | | | | |
| B: Association between ICP trajectories and clinical outcomes in elder patients (adjusted model) | | | | | | |
| Outcomes | Group 1 (ref) | Group 2 | Group 3 | Group 4 | Group 5 | Group 6 |
| **Primary outcomes** |  |  |  |  |  |  |
| All-cause mortality, OR (95% CI) | 0.32 (0.01, 4.19) | 0.09 (0.01, 0.77) | 0.08 (0.01, 0.71) | 0.13 (0.01, 1.20) | 1.01 (0.03, 35.47) | ref |
| P Value | 0.415 | 0.045 | 0.038 | 0.100 | 0.991 |  |
| GCS value at  discharge from ICU, OR (95% CI) | 2.52 (0.17, 38.82) | 1.98 (0.10, 9.52) | 1.14 (0.12, 10.94) | 1.69 (0.07, 7.10) | 1.19 (0.01, 4.27) | ref |
| P Value | 0.489 | 0.988 | 0.900 | 0.749 | 0.297 |  |
| Abbreviations: ICP = intracranial pressure, OR = odds ratio, CI = confidence interval, GCS = Glasgow Coma Score, | | | | | | |
| ICU = intensive care unit. | | | | | | |

| Table S10 Literature review of ICP threshold in adult patients with ABI | | | | | | | | | |
| --- | --- | --- | --- | --- | --- | --- | --- | --- | --- |
| Authors | Year | Journal | Country | Study type | No. | Inclusion | Statistical model | Conclusion | Limitation |
| Donnelly J | 2021 | Br J Anaesth | UK | Retrospective study | 1112 | severe TBI | Pearson correlation, LR | >=20mmHg lasting longer than 13minutes was associated with bad outcome | NA |
| Zeiler FA | 2021 | J Neurosurg Anesthesiol | UK | Retrospective study | 128 | TBI | LR | Above individual epidemiologic ICP threshold had stronger associations with mortality | The heterogeneity of treatment, simplicity of this method, small sample |
| Hawryluk GWJ | 2020 | JAMA Neurol | USA | Retrospective study | 523 | TBI | PCA, ENR | >= 19 mm Hg was associated with mortality | No causal inference, single institution, high rate of loss to follow-up |
| Åkerlund CA | 2020 | PLoS One | Canada | Retrospective study | 227 | TBI | Boostrap | 18 ± 4 mmHg (2 standard deviations) was associated with worse outcomes | No causal inference, confounders, ICP vulnerability might change over time |
| Magni F | 2015 | Stroke | Italy | Prospective study | 55 | SH | LR | Moderate PTDICP30 was associated with unfavorable outcome | Single center, small sample size, no systematic complications |
| Güiza F | 2015 | Intensive Care Med | Belgium | Prospective study | 261 | TBI | LR | >= 20mmHg longer than 37 minutes was associated with worse outcomes | Small sample, changes in ICU management, no casual inference, unmeasured confounders |
| Sorrentino E | 2012 | Neurocrit Care | UK | Retrospective study | 459 | TBI | LR | 22 mmHg for ICP was identified for both survival and favorable outcomes | Heterogeneity of subgroup sizes |
| Chambers IR | 2001 | J Neurosurg | UK | Retrospective study | 291 | SBI | LR | 35 mmHg for ICP was identified for favorable outcomes | NA |
| Abbreviations: ICP = intracranial pressure; ABI = acute brain injury; No. = number; TBI = traumatic brain injury; LR = logistic regression; NA = not available; | | | | | | | | | |
| PCA = principal component analysis; ENR = elastic net regression, SH = subarachnoid hemorrhage; PTD = Pressure-time dose; ICU = intensive care unit; | | | | | | | | | |
| SBI = severe brain injury | | | | | | | | | |

**The RECORD statement – checklist of items, extended from the STROBE statement, that should be reported in observational studies using routinely collected health data.**

|  | **Item No.** | **STROBE items** | **Location in manuscript where items are reported** | **RECORD items** | **Location in manuscript where items are reported** |
| --- | --- | --- | --- | --- | --- |
| **Title and abstract** | | | | | |
|  | 1 | (a) Indicate the study’s design with a commonly used term in the title or the abstract (b) Provide in the abstract an informative and balanced summary of what was done and what was found | Page 2 | RECORD 1.1: The type of data used should be specified in the title or abstract. When possible, the name of the databases used should be included.  RECORD 1.2: If applicable, the geographic region and timeframe within which the study took place should be reported in the title or abstract.  RECORD 1.3: If linkage between databases was conducted for the study, this should be clearly stated in the title or abstract. | Page 2 |
| **Introduction** | | | | | |
| Background rationale | 2 | Explain the scientific background and rationale for the  investigation being reported | Page 3, 4, 5 |  | Page 3, 4, 5 |
| Objectives | 3 | State specific objectives, including any prespecified hypotheses | Page 5 |  | Page 5 |
| **Method5** | | | | | |
| Study Design | 4 | Present key elements of study design early in the paper | Page 5 |  | Page 5 |
| Setting | 5 | Describe the setting, locations, and relevant dates, including  periods of recruitment, exposure, follow-up, and data collection | Page 5 |  | Page 5 |

| Participants | 6 | 1. *Cohort study* - Give the eligibility criteria, and the sources and methods of selection of participants. Describe methods of follow-up   *Case-control study* - Give the eligibility criteria, and the sources and methods of case ascertainment and control selection. Give the rationale for the choice of cases and controls *Cross-sectional study* - Give the eligibility criteria, and the sources and methods of selection of participants   1. *Cohort study* - For matched studies, give matching criteria and number of exposed and unexposed   *Case-control study* - For matched studies, give matching criteria and the number of controls per case | Page 6 | RECORD 6.1: The methods of study population selection (such as codes or algorithms used to identify subjects) should be listed in detail. If this is not possible, an explanation should be provided.  RECORD 6.2: Any validation studies of the codes or algorithms used to select the population should be referenced. If validation was conducted for this study and not published elsewhere, detailed methods and results should be provided.  RECORD 6.3: If the study involved linkage of databases, consider use of a flow diagram or other graphical display to demonstrate the data linkage process, including the number of individuals with linked data at each stage. | Page 6 |
| --- | --- | --- | --- | --- | --- |
| Variables | 7 | Clearly define all outcomes, exposures, predictors, potential confounders, and effect modifiers. Give diagnostic criteria, if applicable. | Page 6 | RECORD 7.1: A complete list of codes and algorithms used to classify exposures, outcomes, confounders, and effect modifiers should be provided. If these cannot be reported, an explanation should be provided. | Page 6 |
| Data sources/ measurement | 8 | For each variable of interest, give sources of data and details of methods of assessment (measurement).  Describe comparability of assessment methods if there is more than one group | Page 5, 6 |  | Page 5, 6 |

| Bias | 9 | Describe any efforts to address potential sources of bias | Page 8 |  | Page 8 |
| --- | --- | --- | --- | --- | --- |
| Study size | 10 | Explain how the study size was arrived at | Page 6 |  | Page 6 |
| Quantitative variables | 11 | Explain how quantitative variables were handled in the analyses. If applicable, describe  which groupings were chosen, and why | Page 7 |  | Page 7 |
| Statistical methods | 12 | 1. Describe all statistical methods, including those used to control for confounding 2. Describe any methods used to examine subgroups and interactions 3. Explain how missing data were addressed 4. *Cohort study* - If applicable, explain how loss to follow-up was addressed   *Case-control study* - If applicable, explain how matching of cases and controls was addressed  *Cross-sectional study* - If applicable, describe analytical methods taking account of sampling strategy   1. Describe any sensitivity analyses | Page 7, 8 |  | Page 7, 8 |
| Data access and cleaning methods |  | .. |  | RECORD 12.1: Authors should describe the extent to which the investigators had access to the database population used to create the study population. | Page 5, 6 |

|  | |  |  |  | RECORD 12.2: Authors should provide information on the data cleaning methods used in the study. | Page 7 |
| --- | --- | --- | --- | --- | --- | --- |
| Linkage | |  | .. |  | RECORD 12.3: State whether the study include  ed person-level, institutional-level, or other data linkage across two or more databases. The methods of linkage and methods of  linkage quality evaluation should be provided. | Page 5, 6 |
|  | **Results** | | | | | |
| Participants | | 13 | 1. Report the numbers of individuals at each stage of the study (*e.g.*, numbers potentially eligible, examined for eligibility, confirmed eligible, included in the study, completing follow-up, and analysed) 2. Give reasons for non- participation at each stage. 3. Consider use of a flow diagram | Page 9, 10 | RECORD 13.1: Describe in detail the selection of the persons included in the study (*i.e.,* study population selection) including filtering based on data quality, data availability and linkage. The selection of included persons can be described in the text and/or by means of the study flow diagram. | Page 8 |
| Descriptive data | | 14 | 1. Give characteristics of study participants (*e.g.*, demographic, clinical, social) and information on exposures and potential confounders 2. Indicate the number of participants with missing data for each variable of interest 3. *Cohort study* - summarise follow-up time (*e.g.*, average and total amount) | Page 8, 9, 10 |  | Page 8, 9, 10 |
| Outcome data | | 15 | *Cohort study* - Report numbers of outcome events or summary measures over time  *Case-control study* - Report numbers in each exposure | Page 10 |  | Page10 |

|  | |  | category, or summary measures of exposure  *Cross-sectional study* - Report numbers of outcome events or summary measures |  |  |  |
| --- | --- | --- | --- | --- | --- | --- |
| Main results | | 16 | 1. Give unadjusted estimates and, if applicable, confounder- adjusted estimates and their precision (e.g., 95% confidence interval). Make clear which confounders were adjusted for and why they were included 2. Report category boundaries when continuous variables were categorized 3. If relevant, consider translating estimates of relative risk into absolute risk for a meaningful time period | Page 10, 11, 12, , 13, 14, 15, 16, 17, 18 |  | Page 10, 11, 12, , 13, 14, 15, 16, 17, 18 |
| Other analyses | | 17 | Report other analyses done— e.g., analyses of subgroups and interactions, and sensitivity analyses | Page 17, 18 |  | Page 17, 18 |
|  | **Discussion** | | | | | |
| Key results | | 18 | Summarise key results with  reference to study objectives | Page 19, 20 |  | Page 19, 20 |
| Limitations | | 19 | Discuss limitations of the study, taking into account sources of potential bias or imprecision.  Discuss both direction and magnitude of any potential bias | Page 23 | RECORD 19.1: Discuss the implications of using data that were not created or collected to answer the specific research question(s). Include discussion of misclassification bias, unmeasured confounding, missing data, and changing eligibility over time, as they pertain to the study being  reported. | Page 23 |
| Interpretation | | 20 | Give a cautious overall interpretation of results considering objectives, | Page 19, 20, 21, 22 |  | Page 19, 20, 21, 22 |

|  | |  | limitations, multiplicity of analyses, results from similar studies, and other relevant evidence |  |  |  |
| --- | --- | --- | --- | --- | --- | --- |
| Generalisability | | 21 | Discuss the generalisability (external validity) of the study results | Page 22, 23 |  | Page 22, 23 |
|  | **Other Information** | | | | | |
| Funding | | 22 | Give the source of funding and the role of the funders for the present study and, if applicable, for the original study on which the present article is based | Page 24 |  | Page 24 |
| Accessibility of protocol, raw data, and programming  code | |  | .. |  | RECORD 22.1: Authors should provide information on how to access any supplemental information such as the study protocol, raw data, or  programming code. |  |

*Reference: Benchimol EI, Smeeth L, Guttmann A, Harron K, Moher D, Petersen I, Sørensen HT, von Elm E, Langan SM, the RECORD Working Committee. The REporting of studies Conducted using Observational Routinely-collected health Data (RECORD) Statement. *PLoS Medicine* 2015; in press.

*Checklist is protected under Creative Commons Attribution ([CC BY](http://creativecommons.org/licenses/by/4.0/)) license.
